# Optimization of endothelial cell growth in a murine in vitro blood–brain barrier model

**DOI:** 10.1002/biot.201100189

**Published:** 2011-12-28

**Authors:** Diane M Wuest, Kelvin H Lee

**Affiliations:** Chemical Engineering and Delaware Biotechnology Institute, University of DelawareNewark, DE, USA

**Keywords:** Astrocytes, Blood-brain barrier, Co-culture, In vitro blood-brain barrier model, Murine brain microvascular endothelial cells

## Abstract

In vitro cell culture models of the blood–brain barrier (BBB) are important tools used to study cellular physiology and brain disease therapeutics. Although the number of model configurations is expanding across neuroscience laboratories, it is not clear that any have been effectively optimized. A sequential screening study to identify optimal primary mouse endothelial cell parameter set points, grown alone and in combination with common model enhancements, including co-culturing with primary mouse or rat astrocytes and addition of biochemical agents in the media, was performed. A range of endothelial cell-seeding densities (1–8 × 10^5^ cells/cm^2^) and astrocyte-seeding densities (2–8 × 10^4^ cells/cm^2^) were studied over seven days in the system, and three distinct media-feeding strategies were compared to optimize biochemical agent exposure time. Implementation of all optimal set points increased transendothelial electrical resistance by over 200% compared to an initial model and established a suitable in vitro model for brain disease application studies. These results demonstrate the importance of optimizing cell culture growth, which is the most important parameter in creating an in vitro BBB model as it directly relates the model to the in vivo arrangement.

## 1 Introduction

The blood-brain barrier (BBB) separates the central nervous system from systemic blood circulation by physical, metabolic, and transport barriers and is formed by complex interactions between several types of cells, including cerebral endothelial cells and perivascular elements [1, 2]. Endothelial cells compose cerebral capillaries and form a physical barrier via tight junction interactions with adjacent endothelial cells [3–5]. Perivascular elements, namely glial cells, pericytes, and basal lamina, support specialization of the endothelial cells and aid in metabolism and nutrient transport.

Advanced characterization of the BBB has enabled development of in vitro experimental methodologies to better study this intricate system and enhance understanding of how and what molecules cross the BBB. A common approach to in vitro BBB models is monolayer growth of cerebral endothelial cells on a porous membrane submerged in culture media in multi-well plates. Monolayer cultures of brain endothelial cells form tight intercellular junctions, and co-culturing with astrocytes (a type of glial cell) and using enhanced media formulations containing specific biochemical agents can improve the formation of endothelial cell tight junctions [5–9]. The formation of a tight endothelial cell monolayer can be quantitatively characterized with the two techniques of transendothelial electrical resistance (TEER) and diffusive permeability of solutes. TEER measures the paracellular resistance of the monolayer to an electrical current, and solute permeability measures the amount of solute detected in the abluminal side of the membrane after administration in the luminal side.

No single optimal in vitro BBB model exists; instead each lab utilizes a unique collection of model parameter set points to explore BBB physiology and model applications [10, 11, 12]. Recent studies have identified improvements in the purity of primary endothelial cell cultures [13, 14] and the induction of endothelial cell barrier properties with astrocytes, pericytes, or media additives [15–17] using a variety of cell species and growth timelines in their model systems. For example, primary cells can be isolated from cows, pigs, rats, or mice and grown on the membrane in the presence of perivascular elements and/or biochemical agents for 1–10 days. Furthermore, to circumvent the high skill level and large amount of time needed to repeatedly isolate primary brain cultures, a number of immortalized endothelial cell lines have been generated from human, cow, rat, and mouse cells. Recently, in vitro models have increasingly employed human cell lines, such as ECV03 and hCMEC/D3 [18–19]. While immortalized cell lines are easier to culture and sustain over multiple experiments, none fully express all of the necessary BBB characteristics that primary cells contain, and they exhibit low TEER.

Endothelial cell growth is arguably the most important variable in an in vitro BBB model because it directly impacts model performance and barrier formation in the attempt to mimic in vivo cell growth. Therefore, research is needed to identify optimal growth conditions, including seeding density and growth duration on the membrane, before performing experiments aimed to test and measure the functionality and physiology of the established barrier. Growth performance depends specifically on species, purity of primary cultures, and presence of supporting inductive molecules. This dependence emphasizes the need for each lab to address growth performance of their selected system on an individual basis.

In this paper, we report the first systematic study and optimization of the tight monolayer formation of primary murine endothelial cells in the absence and presence of primary astrocytes and media additives with daily TEER measurements. First, optimal seeding densities, growth durations, and media-feeding strategies were determined, and then an astrocyte species comparison was conducted. Mouse astrocytes were observed to perform better than rat astrocytes in TEER and sodium fluorescein permeability measurements. Optimizing growth conditions of endothelial cells and astrocytes as well as the exposure time to biochemical agents present in the media improved our model by over 200% compared to an initial model and provides a useful and well characterized in vitro BBB model to apply in studies of brain pathology and treatment. The systematic approach we created constitutes an exemplary method that can be translated to any BBB system because endothelial cell growth optimization is important and applicable to all in vitro BBB models using any source of cells. Although primary cultures using species with larger brains (e.g., pigs, cows) generate more material per isolation procedure and can generate larger TEER and smaller permeability coefficients than primary murine models, many in vivo BBB drug transport studies are performed in murine models and a large selection of murine antibodies exist. Therefore, murine in vitro BBB models are potentially better models to use in the BBB field to best understand drug transport screening efforts.

## 2 Materials and methods

### 2.1 Chemicals and supplies

Percoll was purchased from GE Healthcare (Waukesha, WI, USA). Platelet-poor plasma derived serum was purchased from Biomedical Technologies, Inc. (Stoughton, MA, USA). 8-CPT-cAMP and RO20-1724 (phosphodiesterase inhibitor) were purchased from Biomol (Plymouth Meeting, PA, USA). l-glutamine was purchased from Fisher Scientific (Pittsburg, PA, USA). Fetal bovine serum (FBS), Dulbecco's Modified Eagle medium (DMEM, with l-glutamine, sodium pyruvate, and low glucose), Ham's F-12 nutrient mixture, TrypLE, penicillin-streptomycin-amphotercin (PSA), and Dulbecco's phosphate buffered saline (DPBS, without CaCl_2_ and MgCl_2_) were purchased from Invitrogen (Carlsbad, CA, USA). Human fibronectin was purchased from Millipore (Billerica, MA, USA). Fungin and primocin were purchased from InvivoGen (San Diego, CA, USA). Gentamicin, HEPES sodium salt, fluorescein sodium salt, heparin, bovine serum albumin, puromycin, hydrocortisone, insulin-transferrin-sodium selenite supplement, retinoic acid, poly-l-lysine, and collagen type IV were purchased from Sigma (St. Louis, MO, USA). Collagenase-dispase was purchased from Roche Molecular Biochemicals (Indianapolis, IN, USA). Basic fibroblast growth factor was purchased from R&D Systems (Minneapolis, MN, USA). Type II collagenase and DNase I were purchased from Worthington Biochemical Corp (Lakewood, NJ, USA). Primary rat brain cortex astrocytes were purchased from Lonza (Walkersville, MD, USA). Cell culture inserts for 24-well plates (0.4 μm pore diameter size, transparent PET membrane) were purchased from BD Falcon (Franklin Lakes, NJ, USA). EVOM and EndOhm-6 voltohmmeter system was purchased from World Precision Instruments (Sarasota, FL, USA).

### 2.2 Isolation of murine brain microvascular endothelial cells

This procedure was performed essentially as described previously [20]. All animals were treated according to protocols evaluated and approved by the Institutional Animal Care and Use Committee (IACUC) at the University of Delaware. Briefly, 10 adult wild type male mice (C57BL/6) were euthanized under CO_2_. Forebrains were collected and stored in DMEM on ice. The remainder of the isolation took place under aseptic conditions. The brains were cut sagittally into two haves and rolled on Whatman 3 mm chromatography paper to remove the meninges. The cortices were dissected away and much of the white matter was removed. The cortices were cut up with forceps and completely triturated and digested with 0.69 mg/mL type II collagenase and 37.6 U/mL DNase I in DMEM for 1 h in a 37°C shaker at 250 rpm. The enzyme solution was then diluted in DMEM and centrifuged at 1000 times; *g* for 8 min at room temperature. To remove myelin, the pellet was resuspended in 20% w/v bovine serum albumin in DMEM and centrifuged at 1000 × *g* for 20 min. The pellet was resuspended and further digested with 0.69 mg/mL collagenase-dispase and 28.3 U/mL DNase I in DMEM for 1 h in a 37°C shaker at 200 rpm. The enzyme solution was then diluted in DMEM and centrifuged at 700 × *g* for 6 min at room temperature. The microvessels were separated on a 33% continuous percoll gradient, collected, centrifuged at 1000 × *g* for 10 min, resuspended in 4 mL culture media and plated in two 35 mm Petri dishes coated with collagen type IV (10%) and fibronectin (10%). Cultures were maintained in growth media consisting of DMEM supplemented with 4 μg/mL puromycin, 20% bovine platelet-poor plasma-derived serum, 1 ng/mL human basic fibroblast growth factor, 1 g/mL heparin, 2 mM l-glutamine, and an antibiotic solution (100 U/mL penicillin, 100 μg/mL streptomycin, and 0.25 μg/mL amphotericin). Culture medium was completely replaced every day, and puromycin was removed from the medium on day 4. Cultures were maintained in a 37°C humidified cell culture incubator with 5% CO_2_.

### 2.3 Isolation of mouse astrocytes

Astrocytes isolated from postnatal mice pups were provided by Dr. Davide Trotti (Department of Biochemistry and Molecular Biology, Thomas Jefferson University, Philadelphia, PA, USA) following established techniques [21]. Cells were maintained in DMEM/F12 (1:1) supplemented with 20% FBS, 0.25% gentamicin, 0.2% fungin, 0.2% primocin, and 1% antibiotic solution (100 U/mL penicillin and 100μg/mL streptomycin). Cells were grown on 75 cm^2^ tissue culture flasks at passage 0 and were fed every 3 or 4 days. Cultures were maintained in a 37°C humidified cell culture incubator with 5% CO_2_ for 1–2 wk prior to use in the in vitro BBB model.

### 2.4 Rat astrocyte culture

Rat brain cortex astrocytes were maintained in DMEM supplemented with 5% FBS, 0.005% gentamicin, and 1% antibiotic solution (100 U/mL penicillin and 100 μg/mL streptomycin). Cells were grown on 75 cm^2^ tissue culture flasks at passage 0 and were fed every 3 or 4 days. Cultures were maintained in a 37°C humidified cell culture incubator with 5% CO_2_ for 1–2 wk prior to use in the in vitro BBB model.

### 2.5 Culture growth and in vitro model set up

**Figure 1:**
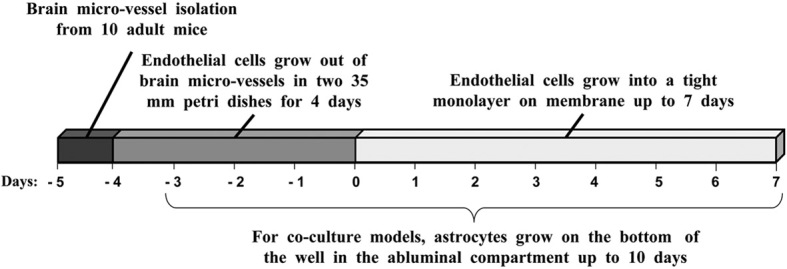
Timeline of in vitro BBB model growth optimization experiments.

After the isolation of the brain microvessels on day 5 (i.e., five days before endothelial cells were seeded on the membrane), endothelial cells grew into a monolayer on the bottom of two 35 mm petri dishes for 4 days. Once the cells were 90–100% confluent they were subcultured onto the membrane of the cell culture insert and grown for up to 7 days (Fig. 1). To facilitate endothelial cell adhesion, the Petri dishes and membranes were coated with collagen type IV (40%) and fibronectin (10%). If a co-culture was established, astrocytes were subcultured from a 75 cm^2^ tissue culture flask and seeded on the bottom of the well in 24-well plates 2 or 3 days before the endothelial cells were seeded onto the membrane (Fig. 1). To facilitate astrocyte adhesion, the well bottoms were treated with 5 μg/cm^2^ of poly-d-lysine for at least 12 h in the culture incubator before astrocyte seeding. Cultures were maintained in the standard endothelial cell growth media (as described in Section 2.2) or an enhanced media. The enhanced media consisted of DMEM/F-12 (1:1) supplemented with 2 mM l-glutamine, 550 nM hydrocortisone, 312.4 μM cAMP, 17.5 μM phosphodiesterase inhibitor, 1 μM retinoic acid, 5 μg/mL insulin, 5 μg/mL transferrin, 5 ng/mL sodium selenite, and an antibiotic solution (100 U/mL penicillin, 100 μg/mL streptomycin, and 0.25 μg/mL amphotericin) [7, 15, 20].

### 2.6 Transendothelial electrical resistance (TEER) measurements

To characterize the formation of a tight endothelial cell monolayer, TEER was obtained by transferring the cell culture insert into the EndOhm-6 chamber and measuring the overall resistance to the current between electrodes. The resistance value of a blank culture insert treated with collagen type IV and fibronectin was subtracted from the total resistance measured, and the resulting value was multiplied by the membrane area to obtain the TEER measurement in OHgr; cm^2^.

### 2.7 Sodium fluorescein permeability measurements

Small molecule permeability measurements across the endothelial cell monolayer from the luminal compartment to the abluminal compartment rely on passive diffusion (molecular movement down a concentration gradient). The flux of the species is equal to the permeability multiplied by the concentration gradient, as shown in Eq. (1) [22].


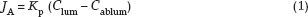
(1)

*J*_A_ is the flux of species A across the endothelial cell monolayer (mol cm^–2^ – s), *K*_p_ is the permeability (cm s^–1^), *C*_lum_ is the luminal concentration, and *C*_ablum_ is the abluminal concentration of species A (mol cm^–3^). In this study, the diffusive flux of sodium fluorescein (376 Da) was obtained by measuring the rate of influx into the abluminal compartment. Prior to the start of permeability studies, culture media from the abluminal compartments were replaced with pre-equilibrated transport buffer (10 mM HEPES, 0.1% w/v bovine serum albumin, 4.5% w/v glucose), and a 1.0 μM sodium fluorescein buffer solution was added to the luminal side of the membrane. Aliquots (100 μL) were removed from the abluminal compartment and the volume was replaced with pre-equilibrated transport buffer every 15 min over 60 min. The diffusive flux of sodium fluorescein of a blank culture insert treated with collagen type IV and fibronectin was subtracted from the total flux measured of the experimental cases, and the permeability coefficient, reported in units of cm s^–1^, was obtained by dividing the volumetric flux by the membrane area [23].

### 2.8 Statistical and error analysis

JMP® v8.0 (SAS Institute Inc., Cary, NC, USA) was used to perform statistical analysis. Statistical evaluation of TEER and permeability data was performed using student's *t*-test with 95% confidence (i.e., differences with a *p*-value of <0.05 were considered to be statistically significant). Standard deviations of the means are displayed in each graph and calculated over two to three independent experiments containing multiple replicates (e.g., 2–3) in each experiment to show reproducibility of results across primary endothelial cell isolation procedures and within experiments.

## 3 Results and discussion

### 3.1 Endothelial cell culture-seeding density and growth duration optimization

**Figure 2:**
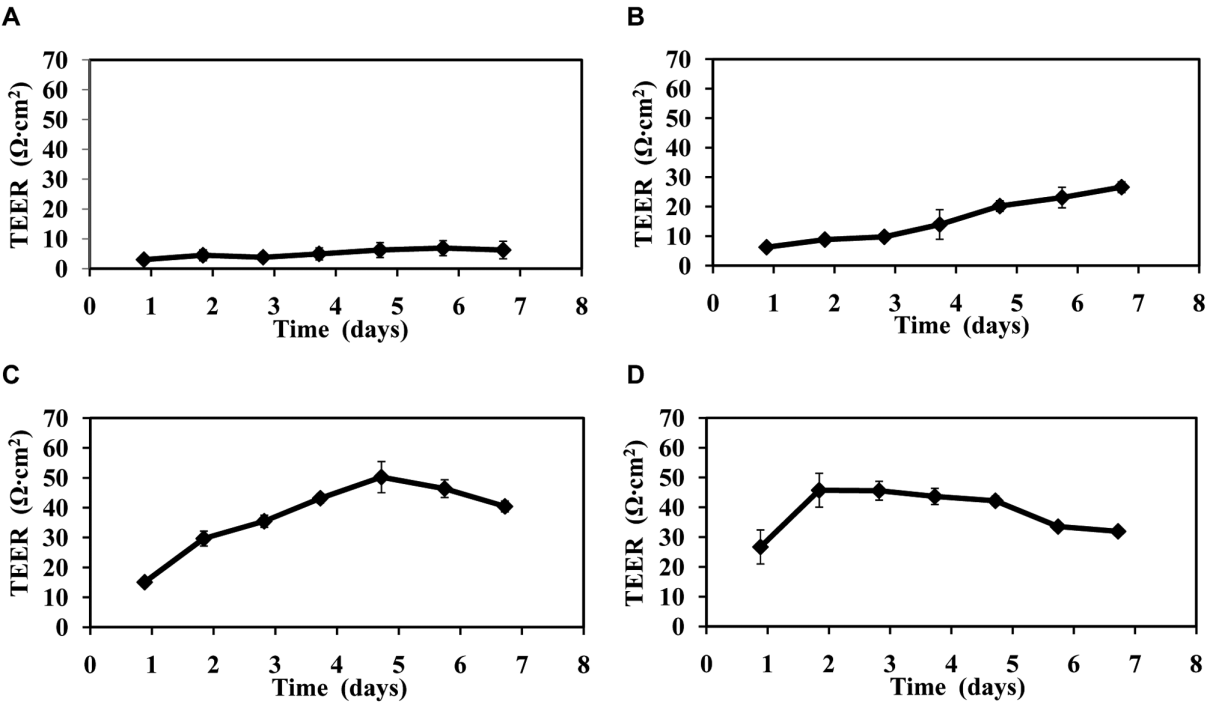
TEER versus time of endothelial cell growth on the membrane in the absence of astrocytes. Profiles from four different endothelial cell-seeding densities: (A) 1 × 10^5^ cells /cm^2^, (B) 2 × 10^5^ cells/cm^2^, (C) 4 × 10^5^ cells/cm^2^, (D) 8 × 10^5^ cells /cm^2^. Error bars show the standard deviation collected over three independent experiments. Culture media was replaced after TEER measurements were taken each day.

The formation of a tight endothelial cell monolayer on the membrane in the absence of astrocytes (i.e., a monoculture of endothelial cells) was monitored with daily TEER measurements over the course of 7 days. Four seeding densities of endothelial cells from 1–8 × 10^5^ cells/cm^2^ were compared (Fig. 2).

The two largest seeding densities, 4 × 10^5^ cells/ cm^2^ and 8 × 10^5^ cells/cm^2^, achieved the tightest endothelial cell monolayer by day 5 or 2, respectively, as demonstrated by reaching TEER of approximately 50 OHgr; cm^2^ (Fig. 2C–D). Subsequent decreasing TEER can be attributed to a loosening of the monolayer, which is likely the result of cells dying and losing the ability to form intercellular tight junctions. Although the cells seeded at 8 × 10^5^ cells/cm^2^ reached their maximum TEER value three days sooner than the cells seeded at 4 × 10^5^ cells/cm^2^, the more favorable seeding density is 4 × 10^5^ cells /cm^2^. Using the 4 × 10^5^ cells/cm^2^ seeding density allows for double the number of cases per mouse brain microvessel isolation procedure from only three additional days of growth on the membrane, whereas seven additional days would be required to obtain the same number of cases using the 8 × 10^5^ cells /cm^2^ seeding density by repeating the entire experiment.

After 7 days of growth on the membrane, the 2 × 10^5^ cells /cm^2^ seeding density achieved a TEER value of about 27 OHgr; cm^2^ (Fig. 2B), and the 1 × 10^5^ cells/cm^2^ seeding density only achieved a TEER value of about 8 OHgr; cm^2^ (Fig. 2A). These data suggest that a minimum number of cells are required to attain a tight barrier in less than seven days using these mouse brain endothelial cells.

### 3.2 Astrocyte growth duration optimization

**Figure 3:**
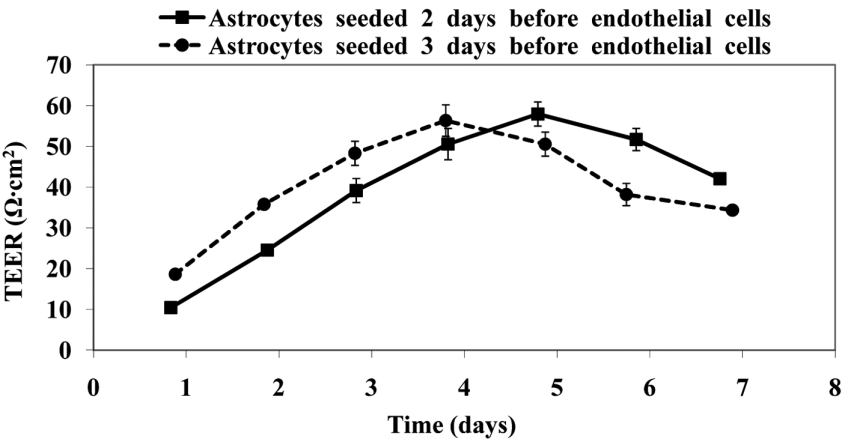
TEER versus time of endothelial cell growth on the membrane in the presence of astrocytes seeded on the bottom of the well. Endothelial cells were seeded at 4 × 10^5^ cells/cm^2^ and astrocytes were seeded at 4 × 10^4^ cells/cm^2^. Error bars show the standard deviation collected over two independent experiments. Culture media was replaced after TEER measurements were taken each day.

The endothelial cell monolayer tightness (using the optimal 4 × 10^5^ cells/cm^2^ seeding density) on the membranes in the presence of astrocytes seeded on the bottom of the well was monitored using daily TEER measurements over the course of 7 days. Astrocytes were seeded at 4 × 10^4^ cells/cm^2^, two or three days before endothelial cells were seeded on the membrane (Fig. 3).

The co-culture with astrocytes seeded two days before the endothelial cells (solid curve in Fig. 3) showed the largest TEER on day 5, whereas the co-culture with astrocytes seeded three days before the endothelial cells (dashed curve in Fig. 3) showed the largest TEER on day 4. Therefore, the astrocytes had their greatest effect on the endothelial cells after seven days of growth in the abluminal compartment. Maximizing TEER on day 5, which occurred when astrocytes were seeded two days before the endothelial cells, is preferred because the endothelial cell monoculture showed the tightest barrier in the absence of astrocytes on this day (Fig. 2C). Studies using two additional astrocyte-seeding densities of 2 × 10^4^ cells/cm^2^ and 8 × 10^4^ cells /cm^2^ confirmed the same trends as seeding at 4 × 10^4^ cells/cm^2^ (data not shown). In all cases, the presence of astrocytes increased the resistance of endothelial cells approximately 10 OHgr; cm^2^ as compared to the endothelial cell monocultures.

### 3.3 Astrocyte-seeding density and media-feeding strategy optimization

**Figure 4:**
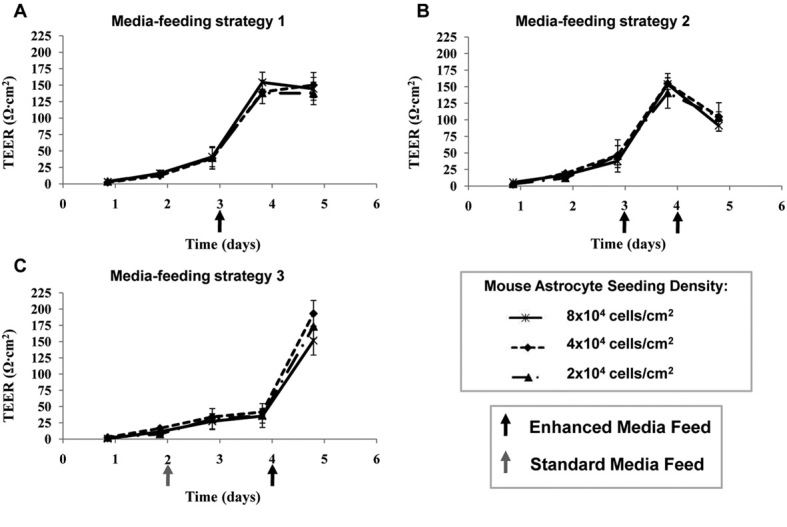
TEER versus time of endothelial cell growth on the membrane in the presence of astrocytes seeded on the bottom of the well. Endothelial cells were seeded at 4 × 10^5^ cells/cm^2^, and astrocytes were seeded two days before endothelial cells were seeded on the membrane. Co-cultures were treated with three media-feeding strategies: (A) Enhanced media feed on day 3, (B) two sequential enhanced media feeds on day 3 and 4, and (C) standard media feed on day 2 and an enhanced media feed on day 4. Error bars show the standard deviation collected over two independent experiments. All media feeds were executed after TEER measurements were taken.

Endothelial cell monolayers treated with three different media-feeding strategies and in the presence of three mouse astrocyte-seeding densities (seeded two days before the endothelial cells) were monitored using daily TEER measurements. Co-cultures were treated with three media-feeding strategies, and three astrocyte-seeding densities from 2–8 × 10^4^ cells /cm^2^ were investigated (Fig. 4). Media-feeding strategy 1 included an enhanced media feed (composition listed in Section 2.5) on day 3 (Fig. 4A), media-feeding strategy 2 included two sequential enhanced media feeds on days 3 and 4 (Fig. 4B), and media-feeding strategy 3 included a standard culture media feed on day 2 and an enhanced media feed on day 4 (Fig. 4C).

The largest average TEER observed in this set of experiments, 195 OHgr; cm^2^ was observed when media-feeding strategy 3 and the 4 × 10^4^ cells/cm^2^ astrocyte-seeding density were used (Fig. 4C). In this set of experiments, larger TEER values were observed when the media was not removed daily (as in Figs. 2 and 3), possibly due to the accumulation of beneficial agents secreted by the astrocytes that tighten the endothelial cell monolayer over time. Furthermore, the drastic improvement in TEER values occurring after the first enhanced media feed can be attributed to the specific biochemical agents present (hydrocortisone, cAMP, phosphodiesterase inhibitor, retinoic acid, insulin, transferrin, and sodium selenite) [7, 15, 20]. Although the mouse astrocyte-seeding density parameter was not statistically significant, the 4 × 10^4^ cells/cm^2^ astrocyte- seeding density achieved the largest TEER and was chosen as optimal.

Media-feeding strategy 3 was the optimal feeding strategy across all astrocyte-seeding densities, achieving the largest TEER on day 5 when the endothelial cells form the tightest barrier. The enhanced media feed was most beneficial when applied one day before the intended measurement day, not two days before (strategy 1) nor over two consecutive days (strategy 2). These results suggest that the effect of the biochemical agents in the enhanced media potentially decreased after one day in culture, and it was surprising to observe that replenishing the enhanced media decreased the TEER. The standard media feed in strategy 3 allowed the cells to maintain healthy growth before the day 4 enhanced media feed. Additional studies using rat astrocytes with the same conditions as in Fig. 4 showed consistent optimal conditions (data not shown).

### 3.4 Astrocyte species optimization

**Figure 5:**
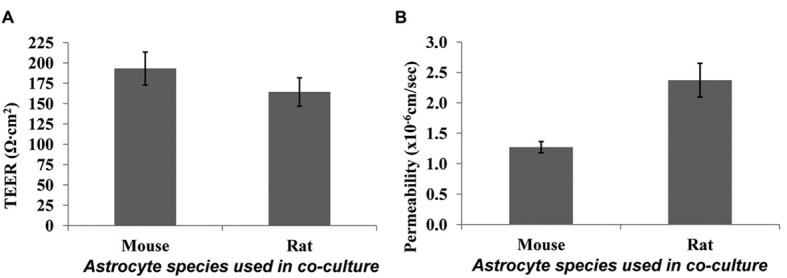
Comparison of endothelial cell monolayers grown in the presence of mouse versus rat astrocytes seeded on the bottom of the well. (A) TEER data; and (B) sodium fluorescein permeability data. Endothelial cells were seeded at 4 × 10^5^ cells/cm^2^, astrocytes were seeded at 4 × 10^4^ cells /cm^2^ two days before endothelial cells were seeded, and all cultures were fed using media-feeding strategy 3. Data was collected on day 5 of endothelial cell growth on the membrane, and error bars show the standard deviation collected over three independent experiments.

Endothelial cell monolayers in the presence of mouse or rat astrocytes seeded at 4 × 10^4^ cells/cm^2^, two days prior were characterized with TEER and sodium fluorescein permeability measurements after five days of growth on the membrane (Fig. 5). Media-feeding strategy 3 was employed, which included a standard media feed on day 2 and an enhanced media feed on day 4.

The endothelial cell monolayer displayed statistically significantly larger TEER (*p* = 0.04) and smaller permeability (*p* = 0.03) in the presence of mouse astrocytes than rat astrocytes (Fig. 5). Therefore, mouse astrocytes had a significantly stronger effect on tightening the endothelial cell monolayer than the rat astrocytes. These results may be explained by an intra-species synergy between the two types of brain cells. Additionally, the rat astrocytes were visually observed to grow more aggressively than the mouse astrocytes.

## 4 Concluding remarks

**Figure 6:**
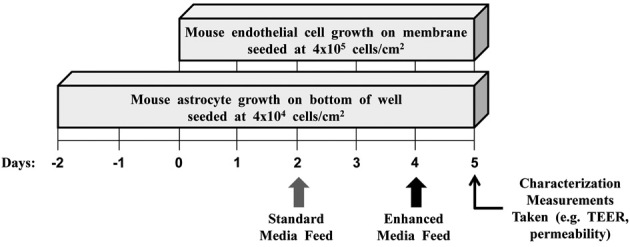
Timeline of optimal conditions for in vitro BBB model application experiments.

We report the first systematic screen of endothelial cell growth conditions and determined optimal growth set points for a murine in vitro BBB model (Fig. 6). Understanding the growth behavior of primary endothelial cells, which is mainly determined by species, seeding density, and growth duration, is important to establish an optimal in vitro BBB model. Additional cell culture enhancements that aid in formation of tight junctions such as co-culturing with astrocytes and introducing biochemical agents into the media can be most effectively optimized after establishing the endothelial cell monoculture set points. As variability is likely to exist in primary endothelial cell isolation yields and model set up, it may be beneficial to conduct an optimization study, such as the one presented here, prior to applying the model. Our work demonstrated a 200% improvement in TEER after implementing optimized set points, achieving a TEER of 200 OHgr; cm^2^ and a sodium fluorescein permeability coefficient of 1.25 × 10^–6^ cm/s, which provides a valuable in vitro BBB model. It has been reported that endothelial cell monolayers are sufficiently tight and applicable for transport studies when displaying TEER in the range 150–200 OHgr; cm^2^ [24, 25]. Comparing TEER and sodium fluorescein permeability results from this primary murine in vitro BBB model to different primary murine models listed in Deli et al. 2005, this model is superior or equivalent [12]. Although disparities among labs make comparisons difficult, this study demonstrates the importance and benefit of dedicating time to optimize of cell growth parameters. The approach presented here for optimizing cell growth is translatable across models using cells cultured from different species and generation types. Careful implementation of optimal model set points should facilitate model consistency and improve success.

## Abbreviations

BBB: blood-brain barrier
DMEM: Dulbecco's Modified Eagle medium
TEER: transendothelial electrical resistance
